# Vital Pulp Therapy in Primary Dentition: Pulpotomy—A 100-Year Challenge

**DOI:** 10.3390/children8100841

**Published:** 2021-09-24

**Authors:** Andreea Igna

**Affiliations:** Pediatric Dentistry Research Center, Department of Paediatric Dentistry, Faculty of Dentistry, “Victor Babeş” University of Medicine and Pharmacy, 300041 Timişoara, Romania; igna.andreea@umft.ro

**Keywords:** primary teeth, pulpotomy, challenges

## Abstract

Pulpotomy has long been the most indicated vital pulp procedure in primary molars with extensive caries. The success of a pulpotomy is highly technique sensitive and it depends upon many factors, such as diagnosis accuracy, caries excavation method, pulp dressing material, quality of the final restoration and operator experience. This paper provides an overview of the pulpotomy procedure in primary teeth over a century, with reference to advances in technique, medication and restoration possibilities and challenges and controversies surrounding the subject as well as future directions.

## 1. Introduction

Dental caries is a major health problem still exhibiting a very high prevalence in 2021 in children all around the globe. Due to various reasons (lack of proper dental education, lack of access to dental care, “silent symptomatology”, etc.), treatment is often initiated when the progression degree has reached a deep, cavitary stage, often with pulp involvement. The main objective of pulp therapy in primary dentition is promoting the health of the teeth and their supporting tissues to maintain the proper functions of the oro-facial complex (mastication, speech, aesthetics) and ultimately to retain the teeth in their position to preserve arch length [1,2,3]. In paediatric dentistry, pulpotomy is a conservative clinical procedure commonly performed in primary molars with extensive caries, which implies removal of the coronal pulp and preservation of the radicular pulp. The rationale is based on the healing ability of the remaining pulp tissue following surgical amputation of the affected or infected coronal pulp [4]. After having achieved haemostasis, the exposed pulp stumps are covered either with a pulp-capping agent that promotes healing or with an agent to fix the underlying tissue [5].

An electronic search of the existing literature on pulpotomy in primary teeth between 1920 and 2021 in the MEDLINE (PubMed), Google Scholar and Cochrane databases was conducted, using the expressions “vital pulp therapy”, “pulpotomy” and “primary teeth”. Selection of the papers was done with consideration for adequate chronologic perspective over the materials and techniques used by dental professionals across the globe. This paper provides a narrative review on the pulpotomy procedure in primary teeth over time, with reference to advances in technique, medication, restoration possibilities, challenges and controversies surrounding the subject, as well as future directions.

### 1.1. Diagnosis Challenges in Primary Teeth

For primary molars affected by deep carious lesions, pulpotomy has been the most commonly indicated vital pulp therapy [6], considering that microorganisms or their toxins may have reached the pulp [7]. The caries removal method can decisively influence the treatment choice: while the use of a high-speed handpiece or laser might result in exposure of a “normal” pulp that would otherwise not be exposed [8], the stepwise caries removal method (two-visit caries excavation) results in fewer pulp exposures [9]. According to the American Academy of Pediatric Dentistry, direct pulp capping is indicated in a primary tooth only when conditions for a favourable response are optimal. Therefore, where bacterial contamination of an otherwise asymptomatic primary tooth is suspected, pulpotomy is considered the procedure of choice. The main indications of pulpotomy are: teeth with extensive caries, no spontaneous pain and no evidence of radicular pathology [1]. However, correlation between symptoms and pulpal status is frequently a challenging task for the paediatric dentist. Care should be taken not to misinterpret a throbbing pain, simulating an irreversible pulp condition, with that associated with an inflamed dental papilla owing to food impaction [10]. A glass ionomer interim temporary restoration placed for 1–3 months prior to vital pulp therapy (VPT) was found to improve accuracy of diagnosing the pulp’s clinical status and subsequent VPT success [11].

### 1.2. Pharmacological Pulpotomy Agents: Which Is the Best Pulp Medicament for Pulpotomised Primary Molars?

Pulpotomy agents evolved along the last century from the action of devitalization (mummification, cauterization) to preservation of the radicular pulp (minimal devitalization, non-inductive) and ultimately to tissue regeneration (reparative, inductive) [12]. The most popular materials that have been used over time, with acceptable results, are: formocresol (FC), ferric sulphate (FS), sodium hypochlorite (SH), calcium hydroxide (CH), calcium-silicate based biomaterials like mineral trioxide aggregate, (MTA) Biodentine^TM^ and bioceramic paste/putty. Some of the afore-mentioned substances (FS and SH) are used in achieving haemostasis (an essential requirement in the pulpotomy procedure), either as stand-alone medication or in combination with other agents. A non-pharmacological approach to gaining haemostasis is pressure with sterile cotton pellets, applied dry or lightly moistened in saline solution [10].

In 1904, Buckley introduced the use of FC, a formaldehyde solution, in the pulp therapy of primary teeth [13]. In 1937, Sweet C.A. modified Buckley’s original solution into a mixture of zinc oxide, eugenol and FC. Although initially the FC pulpotomy technique was done in multiple visits with the objective of complete fixation, mummification and sterilization of the remaining radicular pulp [14], the number of visits was reduced with time. Ultimately, a 5-min dilute FC protocol was established as standard treatment [15]. In this form, FC has been widely used as a pulpotomy medicament across the globe, for a very long time. It has proven to be a forgiving technique, where teeth with partially devitalised pulps were maintained in the arch with chronic, silent inflammation that only sometimes led to abscesses [16,17]. Concerns have been raised over the use of FC in humans, as studies reported its potential for local and systemic side effects [8] like local pulpal inflammation/necrosis, general cytotoxicity, mutagenic/carcinogenic effect, systemic disturbances and immunologic responses [18]. On the other hand, a study by Kahl J., et al. analysed the levels of FC-based pulpotomy agent’s components present in the blood of children that underwent pulpotomy procedures under general anaesthesia and found that, when used in doses typically employed for a pulpotomy procedure, FC poses no risk to children [19]. Concerns have also been expressed about the potential effect of FC on the enamel structure of the permanent successors. Two studies found enamel defects of bicuspids that followed FC pulpotomised primary molars [20,21].

While search for an alternative medicament for primary tooth pulpotomies began, there were authors that recommended a 1:5 dilution of the standard FC solution to be used in the meantime [22]. Glutaraldehyde and ferric sulphate have initially emerged as less toxic FC alternatives. Out of the two, FS has been used more extensively with good clinical and radiographical outcomes. It produces minimal devitalization and preservation of the pulp tissue [15,23,24]. It also exhibits a good haemostatic action, as well as antimicrobial activity. The antibacterial efficacy of ferric sulphate was found to be similar to 0.2% chlorhexidine digluconate on oral microorganisms such as *Staphylococcus aureus*, *Enterococcus faecalis*, *Candida albicans*, *Candida albicans*, *Porphyromonas gingivalis*, *Lactobacillus acidophilus*, *Lactobacillus salivarius*, *Streptococcus mutans*, *Streptococcus sobrinus* and *Aggregatibacter actinomycetemcomitans*, under in vitro conditions [25,26].

While FS shows good overall success on long-term follow-ups, the most common failure of this agent is internal resorption, also seen in FC or glutaraldehyde pulpotomies [23]. However, there are other authors who believe that internal resorption in most cases does not interfere with tooth survival. *Papagiannoulis* L. reported two cases where internal resorption on the 12th month recall had self-repaired using hard tissue on the following recall periods [27].

SH, a common irrigation agent for root canals, also used as a haemostatic agent or for removal of debris and biofilm, proved to be a suitable solution for conducting pulpotomy in primary teeth as well, with evidence of dentinal bridge formation [28]. A 5% SH pulpotomy showed clinical and radiographical success rates similar to FC. The use of SH as an antibacterial agent prior to application of the pulpotomy agent improved the success of CH pulpotomies to equal the success of MTA pulpotomies (for observation up to 12 months) [29].

In 1920, Hermann introduced CH for root canal fillings. Between 1928 and 1930 he studied the reaction of vital pulp tissue to CH to prove that it was a biocompatible material. Since then, CH has been recommended by several authors for direct pulp capping, but it took until the middle of the 20th century until it was regarded as the standard of care [30]. In the following decades, CH has become the gold standard in vital pulp therapy of permanent teeth. When it was introduced in paediatric dentistry, it was presented as an alternative to FC for vital pulp treatment in primary teeth [31]. Placed in permanent teeth, CH results in calcific (dentin) bridge formation, but in primary teeth it seems to cause internal resorption [32]. Though it has been established that the mechanism behind the resorption process is due to the odontoclasts that form in response to CH, the exact process is still to be investigated [33]. Internal resorption rarely causes clinical symptoms and is generally evaluated in radiographic examination. The use of CH in pulpotomy in primary teeth showed less long-term success than the previous materials, namely FC and FS [34].

A 2017 systematic review and meta-analysis of the existing literature on vital pulp therapy in primary teeth concluded that the highest level of success and quality of evidence supported the pulpotomy techniques of MTA and FC for the treatment of deep caries in primary teeth after 24 months [35]. MTA, a calcium silicate-based cement, was introduced by Torabinejad M. in 1993 for endodontic use. Since then, paediatric dentists have successfully employed MTA in a variety of endodontic/restorative applications [36]. In both animal and human studies, MTA showed excellent potential as pulp-capping and pulpotomy medicament, as it is a highly biocompatible material, with regenerative potential and effective induction of dentinal bridge formation [37]. Compared with FC and CH, MTA reduced both clinical and radiological failures, with statistically significant differences at 12 and 24 months [38]. MTA also has an antibacterial effect against *Enterococcus faecalis* and *Streptococcus sanguis*, but not against the anaerobes [39]. A recent study suggests MTA to be a useful material to be placed in contact with the dental pulp, in both infected and uninfected pulp tissue [40]. An in vitro toxicity study comparing several pulpotomy agents (MTA, CH, FS, diluted FC and Buckley’s FC) demonstrated that MTA has the lowest toxicity [41]. MTA used for pulpotomies in primary teeth has been reported to have no adverse effects on the permanent successors as well [42]. The years of clinical experience have revealed some disadvantages of MTA that occur in practice, such as long setting time, potential of discoloration and lengthy procedure [43,44]. Despite these inconveniences, evidence-based studies have pointed towards MTA as the best option yet for pulpotomies in primary molars, followed by Biodentine™, enamel matrix derivatives and laser application or Ankaferd Blood Stopper^®^ (a plant-based haemostatic agent used to control gastrointestinal bleeds) as second choices [45].

Released in 2009 by Septodont, Biodentine™ is a tricalcium silicate cement, similar in composition to MTA. It was designed as a “dentine replacement” material and is highly biocompatible and biologically active, with multiple clinical indications [46]. Studies demonstrated the potential of Biodentine™ to induce odontoblast differentiation from pulp progenitor cells and to form homogenous complete dentinal bridges, with no signs of inflammatory pulp response [47,48,49,50]. Due to its alkaline pH, Biodentine™ seems to exhibit some antimicrobial properties on *Streptoccocus sanguis* and *Streptoccocus salivarius* species, but not on *Streptoccocus mutans* species [51]. A recent review on Biodentine^TM^ confirmed its ability to overcome the major drawbacks of MTA, exhibiting a short initial setting time and superior flexural strength and sealing ability [52]. The solubility of Biodentine™, when tested in acidic environments, was limited and lower than in other water-based cements, like glass-ionomers [53]. A surface remineralization process that produces deposition of an apatite-like structure offers protection against solubility in acidic environments and increases the marginal seal of the material [54]. Therefore, Biodentine™ could be used both as a dentin substitute and for temporary enamel restoration for up to 6 months [55]. Biodentine^TM^ showed significant potential as a pulpotomy agent for regenerative pulpotomies, with similar clinical results as MTA. Both materials proved to be superior to calcium hydroxide, FC or FS as pulpotomy agents in primary dentition [44,52,56].

The mixing and handling characteristics of the powder/liquid systems (like MTA and Biodentine™) are very technique sensitive, produce considerable waste and are time-consuming. Pre-mixed bioceramic materials—EndoSequence Root Repair Material (ERRM; Brasseler USA, Savannah, GA, USA)/TotalFill BC RRM (FKG, La Chaux-de-Fonds, Switzerland), available in the form of syringeable paste or condensable putty)—have the advantages of uniform consistency, lack of waste and fast and precise application (especially the syringeable version). These biomaterials possess the biological properties of calcium-silicate-based cements and are used in VPT procedures with reported success rates comparable to MTA [57,58]. On the other hand, one of the drawbacks of all the afore-mentioned calcium silicate-based cements would be the long setting time. A material that fixes this inconvenience is TheraCal (Bisco Inc., Schaumburg, IL, USA), a light-curing resin-modified tricalcium silicate. Despite exhibiting a higher calcium releasing ability and lower solubility than either MTA and calcium hydroxide, it performs inferiorly to MTA and Biodentine^TM^ as a pulpotomy agent [59]. There are also authors advising against use of resin-containing TheraCal in direct contact with the pulp, because of its toxicity to the pulp tissue [60].

Other materials exhibiting bioactive and regenerative potential that have been studied for the purpose of preserving vitality of the radicular pulp are: osteogenic protein, bone morphogenic protein, freeze-dried bone, bioactive glass, platelet rich plasma, enamel matrix derivative gel, nanohydroxyapatite paste and collagen particles impregnated in antibiotics. As the search for more biocompatible materials to be used in vital pulp therapy is on, here are some natural alternatives that have been investigated for this purpose: propolis, *Curcumin longa* (turmeric), *Aloe vera*, Ankaferd Blood Stopper^®^, an antioxidant mix, *Thymus vulgaris* (thyme), Allium sativum oil (garlic oil) and *Nigella sativa* oil. Given the multitude of alternatives and the scarcity of studies on the topic of natural pulpotomy medicaments, there is insufficient evidence to support the efficacy of their use in primary teeth, and further robust studies are required before such alternative medicaments should be used in clinical practice [61].

### 1.3. Non-Pharmacological Pulpotomy Techniques: Is There a “Chemical-Free” Way to Perform a Successful Pulpotomy?

A retrospective study by Hui-Derksen E.K. evaluated pulpotomies completed without the use of a fixative, preservative or astringent agent. Following haemostasis with cotton pellets, a reinforced zinc-oxide-eugenol base was placed in the pulp chamber, directly onto the pulp stumps, and the final restoration was completed using a stainless-steel crown/amalgam. Overall clinical and radiographical success rates were 94%, the most frequently observed pathologic pulpal response being furcation radiolucency (~4%) [62].

There are also other non-pharmacological treatment modalities used for the pulpotomy procedure, namely electrosurgery and laser. Electrosurgery involves cutting and coagulating the tissues using high-frequency radio waves that pass through tissue cells [63]. The first electrosurgical pulpotomies were attempted in 1983 and 1987 in primate teeth, with conflicting results [64,65,66,67]. Newer studies advocate the use of electrosurgery as a viable alternative to FC, as it produces less histopathological reaction to the pulp tissue and shows similar success rates [17,67]. The advantages of the electrosurgical procedure are speed and lack of pharmacological agents that may produce undesirable systemic effects, while a negative consideration is lateral heat production [17]. The use of laser therapy in pulpotomies was first reported by Shoji S. et al. in 1985 [68], who used a carbon dioxide laser. Since then, various laser types have been used in paediatric dentistry in multiple procedures. Due to their versatility, two types of lasers are more frequently used by paediatric dentists, Er:YAG and Er,Cr:YSGG, since they can be used in both hard and soft tissues [69]. Their therapeutic benefits include haemostasis, sterilization and accelerated pulpal wound healing [70]. A 2018 meta-analysis of studies regarding laser pulpotomy in primary teeth showed clinical and radiographic success rates comparable to other pulpotomy techniques, including MTA and FC [71]. Laser pulpotomy was also found to be superior in terms of operating time, patient cooperation, ease of use and pain. On the other hand, a major draw-back of the routine use of electrosurgical or laser pulpotomy would be the cost of the equipment [72].

### 1.4. Factors That Influence the Success of a Pulpotomy

Beside the biological effect of the material used as a pulpotomy agent, the success of the procedure depends on other factors related to diagnosis, technique, final restoration and the operator’s experience. Caries topography and extension can play a role in the treatment decision-making. Primary teeth with proximal carious lesions extending more than 50% through the dentine thickness appear to have more extensive inflammatory pulpal changes than teeth with occlusal caries of a similar depth. An intra-operatory differential diagnosis may be necessary. Furthermore, if caries extends beyond the cemento-enamel junction, it is better to avoid pulpotomy altogether. Obtaining a complete seal of the vital pulp from the oral environment is an essential requirement for a pulpotomy. Complete caries removal should precede opening of the pulp chamber; bacterial contamination of the pulp during caries removal or bacterial infiltration at the tooth-restoration interface can compromise the success of the procedure. Adequate rubber dam isolation is important, especially during pulpotomy in mandibular primary molars, as well as obtaining a good coronal seal of the final restoration. Either intra-coronal restoration or a stainless-steel crown (SSC) may be adequate to achieve a good marginal seal for single-surface (occlusal) restoration on a primary tooth with a life-span of two years or less, whereas for multi-surface restorations (i.e., occlusal-proximal), SSCs are the treatment of choice. The restoration influences the pulp therapy’s long-term results the most. Unfortunately, dentists’ choice of restorative materials in a paediatric dental setting are greatly impacted by patient cooperation. In most cases, younger children tend to be less cooperative than older ones. More invasive procedures typically result in worse behaviour outcomes and generally cooperation declines through the course of the appointment [73,74,75,76]. Thus, temporization of the final restoration is often necessary. Frequently used temporary restorative materials are zinc-oxide-eugenol cement (IRM), glass-ionomer cement (GIC), resin-modified-glass-ionomer cement (RMGIC) or compomers. Biodentine™ could be used both as a dentine substitute and a temporary restoration for up to 6 months. [55]. However, immediate final restoration is desirable whenever possible. The most effective long-term restoration for pulpotomised primary teeth has been shown to be a stainless-steel crown (SSC) due to its good sealing and full coverage [1]. Higher success rates using a SSC were reported when compared with IRM, RMGI or composite restorations. Full coverage of primary teeth that underwent VPT seems to be the most important factor for further survival of the teeth, especially in young children, when the tooth is expected to be preserved for more than 2 years in the mouth [73,77,78]. The clinician’s experience in the field seems to also be a determining factor for the success rate of primary teeth treated using pulpotomy. A retrospective cohort study carried out at the Department of Pediatric Dentistry of Taipei Chang Gung Memorial Hospital disclosed that when the pulpotomy for primary molars was performed by less-experienced resident doctors, a reduced overall success rate was registered [79].

### 1.5. Is Pulpotomy the Past and the Future for the Treatment of Primary Molars with Extensive Caries?

As previously stated, the traditional approach for primary molars with extensive caries implies, in most cases, an indication for pulpotomy, based on the rationale that pulp inflammation precedes exposure [6,7]. However, the questions that arise today are: “Is there another way?” and “Can we obtain the same result is a less invasive way?”. As paediatric dentists, we are concerned equally by providing the best treatment in the least traumatic way for the child. All efforts should be made to avoid pulpal exposure when treating deep carious lesions [4]. In the light of the evidence that is getting stronger on biomaterials and their multiple indications in primary teeth, pulpotomy may be an overrated treatment option. There are more and more “voices” recently stating that there is no justification for discarding direct/indirect pulp capping or indirect pulp treatment (IPT) in favour of pulpotomy in certain clinical situations [9,35,80,81,82,83]. Actually, recent evidence suggests that IPT (selective removal to soft dentin) is preferred over the traditional pulpotomy [4], with reported success rates of 90% and above [84,85]. On the other hand, there is increasing evidence that the long-disputed Hall technique (a simplified method of managing carious primary molars using preformed metal crowns, cemented with no local anaesthesia, caries removal or tooth preparation) is effective in the long-term, showing more favourable outcomes for pulpal health and restoration longevity than conventional restorations [86] and is even more cost effective compared to the pulpotomy procedure [87]. A 2020 report [88] identified that although the Hall technique is recognised, it is not being used, by an outright majority of paediatric dentists across the globe; barriers such as lack of training, perception as substandard dentistry and perceived lack of evidence reduced its use. These facts point towards the need for taking a leap of faith in embracing the less invasive alternatives of treating deep carious lesions in primary teeth, whenever possible.

## 2. Conclusions

In conclusion, the success of a pulpotomy procedure is highly technique sensitive and it depends upon many factors. To start with, an accurate diagnosis at the time of treatment, although frequently a challenging task in primary teeth, is yet an essential requirement. The type and quality of the restoration also influences the success rate of a pulpotomy, as well as the clinician’s experience. The knowledge acquired in the last 100 years allowed scientists to have a better understanding of the biological processes behind the interactions of living tissues with dental materials; important steps have been made in finding materials that are more biocompatible, less toxic and with far less side effects. Minimally invasive treatments have also become more popular and backed by science as alternatives. Although significant progress was registered, the most recent systematic reviews and meta-analyses emphasize the need for further high-quality research in this area, based on uniform standards, to clarify the controversies that still surround this subject.
